# Biodiversity of Demersal Fish Communities in the Cosmonaut Sea Revealed by DNA Barcoding Analyses

**DOI:** 10.3390/genes15060691

**Published:** 2024-05-26

**Authors:** Hai Li, Xing Miao, Rui Wang, Yuzhuo Liao, Yilin Wen, Ran Zhang, Longshan Lin

**Affiliations:** 1 Laboratory of Marine Biodiversity Research, Third Institute of Oceanography, Ministry of Natural Resources, Xiamen 361005, China; lihai@tio.org.cn (H.L.); miaoxing@tio.org.cn (X.M.); wangrui@tio.org.cn (R.W.); jukcheuk@stu.xmu.edu.cn (Y.L.); m210200678@st.shou.edu.cn (Y.W.); zhangran@tio.org.cn (R.Z.); 2 College of Ocean and Earth Sciences, Xiamen University, Xiamen 361102, China; 3 College of Marine Sciences, Shanghai Ocean University, Shanghai 201306, China

**Keywords:** Antarctic fishes, *COI*, cryptic species, Southern Ocean, species identification

## Abstract

The Cosmonaut Sea is one of the least accessed regions in the Southern Ocean, and our knowledge about the fish biodiversity in the region is sparse. In this study, we provided a description of demersal fish diversity in the Cosmonaut Sea by analysing cytochrome oxidase I (*COI*) barcodes of 98 fish samples that were hauled by trawling during the 37th and 38th Chinese National Antarctic Research Expedition (CHINARE) cruises. Twenty-four species representing 19 genera and 11 families, namely, Artedidraconidae, Bathydraconidae, Bathylagidae, Channichthyidae, Liparidae, Macrouridae, Muraenolepididae, Myctophidae, Nototheniidae, Paralepididae and Zoarcidae, were discriminated and identified, which were largely identical to local fish occurrence records and the general pattern of demersal fish communities at high Antarctic shelf areas. The validity of a barcoding gap failed to be detected and confirmed across all species due to the indicative signals of two potential cryptic species. Nevertheless, DNA barcoding still demonstrated to be a very efficient and sound method for the discrimination and classification of Antarctic fishes. In the future, various sampling strategies that cover all geographic sections and depth strata of the Cosmonaut Sea are encouraged to enhance our understanding of local fish communities, within which DNA barcoding can play an important role in either molecular taxonomy or the establishment of a dedicated local reference database for eDNA metabarcoding analyses.

## 1. Introduction

The Antarctic Circumpolar Current (ACC), which is driven by strong westerly winds, encircles the entire Antarctic continent and forms a thermal obstacle by substantially isolating lower-latitude warmer waters from higher-latitude colder waters [1]. Antarctica is geographically separated from other continents by large abyssal basins and great distances [2]. Therefore, the Southern Ocean that surrounds the Antarctic continent represents one of the most unique and extreme environments globally due to subzero temperatures and the widespread presence of sea ice [3]. The environmental characteristics and patterns in the Southern Ocean have been quite stable for more than 20 million years [4], which not only poses great eco-physiological challenges for marine organisms inhabiting Antarctic seas [3] but also allows for the independent evolution of inimitable and well-adapted endemic lives [5]. As a result, fewer than 400 fish species, approximately 2% of the fish species diversity worldwide [6], have managed to successfully colonise Antarctic waters [3].

The fish fauna of the Southern Ocean is dominated by a single group with a high degree of endemism, the perciform suborder Notothenioidei, particularly in the shelf and upper slope areas [7,8]. Most notothenioid fish belong to five families: Nototheniidae, Channichthyidae, Artedidraconidae, Bathydraconidae and Harpagiferidae [7]. Nonnotothenioid fish species living in Antarctic waters mostly belong to typical deep-sea groups such as zoarcids, liparids, macrourids and myctophids, whose distribution is mainly restricted to the lower slope and the abyssalpelagic layer. In particular, the diversity of demersal ichthyofauna varies among ice-free zones, seasonal zones and high Antarctic zones, with a latitudinal shift in species composition [2]. In general, Antarctic fish fauna show an endemism to a very large extent, with nearly 90% of Antarctic fish species restricted to the Southern Ocean [9]. Considering the impact of climate change as well as associated environmental alterations [10], the endemic feature of Antarctic fishes raises concerns about their vulnerability when they are exposed to multiple stress factors, such as increasing water temperature, decreasing salinity and oxygen level, habitat loss and ocean acidification [2]. Meanwhile, as the most speciose vertebrate group, fish are an integral component of ecological networks and have been regarded as effective sentinels of environmental alterations driven by climate change [11]. In the Antarctic marine ecosystem, most Antarctic fishes link small invertebrates of lower trophic levels and top predators; thus, their underlying vulnerability to ecological variation is of significant importance [2] and may serve as a useful indicator of climate change. Therefore, holistic knowledge of Antarctic fishes, particularly their biodiversity, can facilitate our understanding of the impact climate change has on the Southern Ocean ecosystem [6,12].

Although the fish fauna in Antarctic waters was once suggested to be fairly well known after a century of research [9], not all taxa have been completely revealed, as new species—*Bathyraja* sp. Ishiyama, 1958 (cf. *eatonii*) identified in 2008 [13] and *Pogonophryne favosa* Balushkin & Korolkova, 2013, described in 2013 [14]—and cryptic species—for instance, a member of genus *Macrourus* Bloch, 1786, discovered in 2010 [15] and candidates in *Gymnoscopelus bolini* Andriashev, 1962, *Lampanyctus achirus* Andriashev, 1962 and genus *Bathylagus* Günther, 1878, as revealed in 2018 [16]—are being found continuously. Furthermore, the species richness curve has not yet reached an asymptotic level from a historical perspective, suggesting that the species richness might still be underestimated, as several taxa and a number of areas have not been completely investigated [17]. Even in those taxa that have already been defined, some families, such as Rajidae, Muraenolepidida and Harpagiferidae, still need thorough taxonomic revision due to insufficient information on detailed morphological diagnoses or misidentifications in scientific records [17]. All these bottlenecks in respect to the systematics and biodiversity of Antarctic fishes mainly come from the inherent limitations of conventional morphological taxonomy systems, which are built on external visible morphological diagnoses and countable meristic features [18,19] and thus rely heavily on expert knowledge of taxonomy, systemics, natural history, biology, ecology and biogeography [20]. However, Antarctic fishes have tremendous variability in body shape, scale size, colour type and count and pattern of fin ray [21], and they also show significant phenotypic changes during different developmental stages [6,22]. In addition, sibling species may share similar morphological characteristics [23,24]. Therefore, an accurate and effective way to discriminate and identify Antarctic fishes is urgently needed, and DNA barcoding could be adopted as an alternative solution relative to traditional classification systems [25,26]. Since its initial successful use in the Scotia Sea [27], DNA barcoding analyses have been performed in a lot of Antarctic waters and across various fish taxa [12,15,26,28] and have demonstrated to be a robust tool for species discrimination and identification of Antarctic fishes.

The Cosmonaut Sea is a marginal sea northwest of Enderby Land in East Antarctica between the Cooperation Sea and Riiser-Larsen Sea [29], with relatively little impact from anthropogenic activities such as scientific research and commercial fishing [5,17], which could be the reason for the insufficient biological data for the region [30]. To date, only the pelagic fish community of the Cosmonaut Sea has been reported [31,32], while the diversity of demersal ichthyofauna remained obscure until it was characterised by morphological taxonomy recently [33].

In this study, DNA barcoding was adopted as a main approach for species classification to reveal demersal fish diversity of the Cosmonaut Sea and to determine whether these communities differ from those of typical demersal ichthyofauna inhabiting high-latitude Antarctic ice zones. Our findings can provide valuable elementary data for comprehensive ecosystem evaluation and contribute an important scientific reference for conservation and management.

## 2. Materials and Methods

### 2.1. Specimen Collection and Morphological Identification

**Figure 1:**
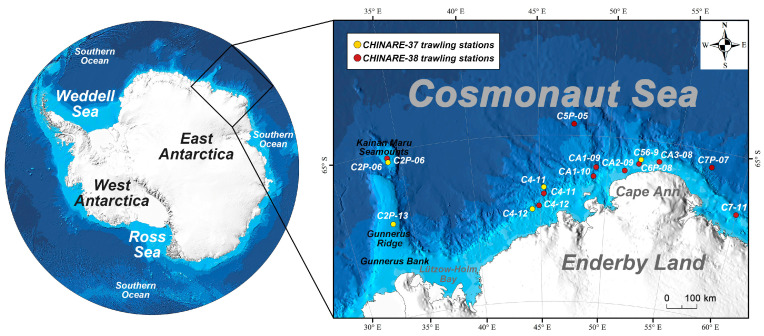
Trawling stations in the Cosmonaut Sea during 37th and 38th CHINARE cruises.

In addition to two fish samples that were obtained as bycatch in stations C7P-07 and C5P-05 by a krill trawl net (8 m^2^ size; 5 mm mesh size), all fish caught were sampled from the Cosmonaut Sea by a triangular bottom trawl net (2.2 m wide, 0.65 m high and 6.5 m long; 20 mm mesh size) on the R/V XUELONG 2 icebreaker during the 37th and 38th Chinese National Antarctic Research Expedition (CHINARE) cruises conducted in 2021 and 2022, respectively (Figure 1). The bottom trawl net was hauled at a speed of 2 knots for 15 min, except for 5 min at station CA3-08 according to the bathymetric topography of the local seabed. Krill trawl net was hauled at a speed of 2 knots for 30 min at a depth of 250 m. All fish samples were roughly categorised and tentatively identified to the finest taxonomic level possible by checking the morphological diagnoses described in Fishes of the Southern Ocean [21] and matching the latest checklist of notothenioid fish species [34] and non-notothenioid fish species [17]. Other pieces of literature were also referred to when resolving the taxonomic identity of a Muraenolepididae member [35]. Muscle tissue samples for molecular analysis were stored in 95% ethanol (Sinopharm Chemical Reagent Co., Ltd., Shanghai, China) following the Barcode of Life protocol for fishes [36]. Species identifications were first conducted onboard and further verified in the laboratory through dedicated morphological examination, and the two rounds of morphological examinations were all implemented by qualified personnel of marine biological monitoring. Both morphologically ambiguous and unambiguous specimens were used for further molecular taxonomic analysis. Voucher specimens were then counted, weighed and fixed in 95% ethanol (Sinopharm Chemical Reagent Co., Ltd., Shanghai, China) at −80 °C onshore in the specimen repository of Third Institute of Oceanography, Ministry of Natural Resources for long-term preservation. A total of 98 specimens were collected during the 37th and 38th CHINARE cruises and used for DNA extraction and PCR amplification.

### 2.2. DNA Extraction, Amplification and Sequencing

Genomic DNA was isolated from muscle tissue near dorsal fin employing a TIANamp Marine Animals DNA Kit (TIANGEN Biotech Co., Ltd., Beijing, China) according to the manufacturer’s protocol. The concentration of template DNA was adjusted to approximately 2.5 to 10 ng/µL prior to amplification. Partial fragments of the mitochondrial cytochrome oxidase I (*COI*) gene, with approximate lengths of 680–690 bp, were amplified using the universal barcoding primers FishF1 and FishR1 [37]. Polymerase chain reaction (PCR) was running in a 25 μL volume that consisted of 2.5 mM MgCl_2_, 0.1 mM dNTPs, 0.25 U of *Taq* polymerase (Takara Biomedical Technology Co., Ltd., Beijing, China), 0.2 μM each forward/reverse primer and 1 μL of DNA template. The thermocycle began with a first step for 4 min at 95 °C; then turned to 35 cycles of 0.5 min at 94 °C, 0.5 min at 52 °C and 0.5 min at 72 °C; and finished with a final step for 10 min at 72 °C. Negative controls were set in all amplifications to detect potential contamination. PCR yields were inspected on 1.5% agarose gels. Final products were directly sequenced using an ABI 3730 capillary sequencer (Applied Biosystems, Foster City, CA, USA) using the BigDye Terminator Sequencing Kit (Applied Biosystems, Foster City, CA, USA) and following the manufacturer’s instructions.

### 2.3. Species Discrimination, Identification and Occurrence Comparison

The sequences were assembled and viewed in Sequencher v4.1.4 [38] and aligned using Clustal W multiple algorithms [39]. Ambiguous sequences and primer binding sites were trimmed after alignment, and final obtained *COI* fragments had a length of 652 bp. The identity of all obtained *COI* fragments was verified by a BLAST [40] search in GenBank (BLASTn, megablast algorithm), comparing the match higher than 98%. Sequences with a match lower than 98% hit against the GenBank database were treated as unidentified. Two distinct molecular species delimitation approaches were adopted to distinguish taxonomic units from the *COI* dataset. The first method, assembly species by automatic partitioning (ASAP), is a new method for highly efficient species partitioning from single-locus sequence alignments [41]. We conducted the *COI* gene fragment alignment through an online tool (https://bioinfo.mnhn.fr/abi/public/asap/asapweb.html, accessed on 17 October 2023) with default settings. The second method, Bayesian phylogenetics and phylogeography (BPP), is a Bayesian Markov chain Monte Carlo (MCMC) program for analysing DNA barcode sequence alignments under the multispecies coalescent model (MSC), which can precisely discriminate sequences at the species level without support from defined distance thresholds in advance and thus brings great improvements to DNA barcoding analyses [42,43]. All obtained *COI* sequences were deposited in GenBank under the accession number PP218555-PP218652.

The most up-to-date fish occurrence records in the Cosmonaut Sea, although scattered, can be best derived from Duhamel et al. (2014), who integrated various national and international data sources, scientists involved in fish surveys, collections of museums and polar institutes and well-referenced published lists of stations and catches [17]. After all sequences were discriminated and identified, the corresponding fish occurrence data in the Cosmonaut Sea were used for comparison with records included in the book chapter across various taxonomic levels.

### 2.4. Genetic Divergence and Phylogenetic Analysis

As suggested by jModeltest 2 [44], pairwise genetic divergent levels were calculated using the Kimura two-parameter (K2P) distance model [45], and neighbour-joining (NJ) trees of K2P distances with 5000 bootstrap replications were also drawn to generate graphic indications of the divergence between species using MEGA X software [46]. The DNA barcoding gap was calculated for all 24 species by comparing the pairwise interspecific genetic distance and pairwise intraspecific genetic distance, according to the criterion of a mean interspecific variability at least 10 times greater than the mean intraspecific genetic distance [47].

Phylogenetic analysis was implemented with all obtained sequences to visualise the relationship among distinct taxonomic units determined by the ASAP and BPP methods. SMS [48] online execution (http://www.atgc-montpellier.fr/sms, accessed on 17 October 2023) was taken to choose the most appropriate model of nucleotide substitution under the Akaike information criterion before conducting phylogenetic analysis. Bayesian analysis was implemented using MrBayes v.3.2 [49]. Parameters for BEAST were inputted in BEAUti 1.10.4 selecting a Coalescent Model with Speciation Yule Process, uncorrelated relaxed clock model, TN93 substitution model and invariant site heterogeneity model with a chain length of 500,000,000 iterations, sampling every 500,000 generations, for the Markov chain Monte Carlo (MCMC) analysis [50]. For maximum likelihood analysis, PhyML 3.0 [51] online tool (https://www.atgc-montpellier.fr/phyml, accessed on 17 October 2023) was chosen following automatic model selection by SMS under the Akaike information criterion. We used NNI as a tree improvement for tree searching and the fast likelihood-based parameter aLRT SH-like for branch support. Majority rule consensus trees were rebuilt after discarding a burn-in of 250 and visualised with FigTree v.1.4.4 (https://tree.bio.ed.ac.uk/software/Figtree). The results of ASAP and BPP were also integrated into the consensus tree for visualisation and comparison.

## 3. Results

### 3.1. Sequence Information

All mitochondrial *COI* barcode fragments were successfully amplified using aforementioned primers. Low-quality sequences such as double peaks, short fragments or background noise were not detected. The full length of the amplified barcode fragments after alignment was 652 bp. Abnormalities such as insertions, deletions or stop codons were not found in the aligned sequences, implying that all barcode alignments were in line with functional mitochondrial *COI* sequences [37]. Among the 652 base sites, 273 were polymorphic, and 53 were parsimony informative. The average base composition was A = 22.25%, C = 29.30%, G = 18.42% and T =30.02% on average, with a slight bias against G and A.

### 3.2. Morphological and Molecular Species Identification and Occurrence Comparison

Most voucher specimens were adult fish in complete shape and could thus be discriminated and inspected. However, there existed also some incomplete specimens and juvenile fish samples, which hindered accurate morphological identification. Thus, although all samples were identified at the finest classification level we could, some could be assigned to only the genus level. In total, 28 morphological species belonging to 19 genera and 11 families were successfully identified according to main morphological diagnoses, including Artedidraconidae (2 species, 3 specimens), Bathydraconidae (5 species, 20 specimens), Bathylagidae (2 species, 3 specimens), Channichthyidae (2 species, 2 specimens), Liparidae (1 species, 1 specimen), Macrouridae (4 species, 34 specimens), Muraenolepididae (2 species, 2 specimens), Myctophidae (3 species, 11 specimens), Nototheniidae (2 species, 18 specimens), Paralepididae (2 species, 2 specimens) and Zoarcidae (2 species, 2 specimens), with Macrouridae being the most specimen-rich family and Bathydraconidae forming the most species-rich family (Table 1). Among the 28 morphological species, 21 species were identified to species level successfully, while 7 species were assigned at the genus level only, namely, *Bathylagus* sp., *Coryphaenoides* Gunnerus, 1765 sp.1, *Coryphaenoides* sp.2, *Notolepis* Dollo, 1908 sp.1, *Notolepis* sp.2, *Pogonophryne* Regan, 1914 sp.1 and *Pogonophryne* sp.2.

As indicated by the book chapter, at least 29 species belonging to 12 families, including Artedidraconidae, Bathydraconidae, Bathylagidae, Channichthyidae, Liparidae, Macrouridae, Muraenolepididae, Myctophidae, Nototheniidae, Paralepididae, Rajidae and Zoarcidae, inhabit the Cosmonaut Sea. Our results verified the existence of thirteen fish species in the Cosmonaut Sea but missed the other seventeen species. However, the fish fauna revealed by our DNA barcoding analyses overlap with historical records across different taxonomic levels to a large extent. At the family level, fish groups were highly identical (92.31%) between our results and existing data except for the absence of Rajidae in our fish catch. At the genus level, half of taxonomic units (50%) in the historical records were shared by CHINARE results. At the species level, almost half (44.83%) of the fish species with occurrence information could still be found on both sides (Table 2).

All obtained fragments were then used to validate species information, which disentangled the consistencies between morphologically determined species and vouchered references. DNA barcode sequences were all successfully assigned to the genus or species level, with the consensus robustness of all fragments determined by alignment through a BLAST annotation in GenBank. Seventy-two morphological identification results (73.47%) matched BLAST searching in the GenBank database with at least 99% similarity, supporting the consistency of species information provided by the two distinct taxonomic approaches. Meanwhile, five other specimens previously identified at the genus level (5.10%) were further assigned to the species level. However, twenty-one morphological identifications (21.43%) were shown to be invalid (Table 1). In particular, the sequence of morphological identification of *Muraenolepis microps* Lönnberg, 1905 did not match any BLAST annotations well except for a *Notomuraenobathys microcephalus* (Norman, 1937) reference. However, the matched reference contained only a 499 bp sequence, which was 153 bp shorter than ours. The authenticity of BLAST annotation for this species was further confirmed after dedicated morphological re-examination of the fish sample based on key morphological characters (Figure S1) [35].

DNA identification (ASAP) was capable of detecting a barcode gap among barcode alignment sequences and implied that 98 sequences formed 25 taxonomic units, among which *P. scotti* Regan, 1914, *Artedidraco shackletoni* Waite, 1911 and *A. skottsbergi* Lönnberg, 1905 consisted of a putative taxonomic unit together, while both *N. coatsorum* Dollo, 1908 and *B. antarcticus* Günther, 1878 split into two putative taxonomic units. However, the phylogenetic trees of BPP analysis suggested that all analysed barcode alignment fragments formed 24 putative species, which almost matched fairly well with the BLAST annotations utilising vouchered sequences existing in the GenBank database. Taking the potential impact insufficient count of most studied species into account [12], the BPP outcome was adopted as the ultimate result.

**Figure 2:**
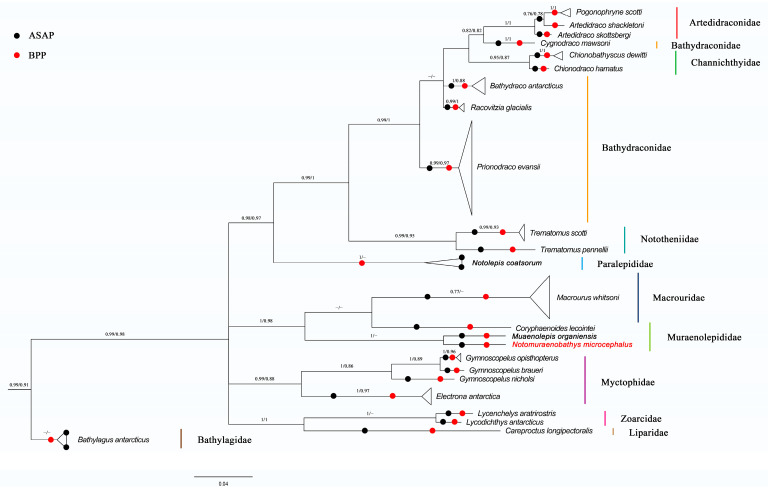
The *COI* phylogenetic tree based on Bayesian inference for demersal fish of the Cosmonaut Sea, obtained with MrBayes, with the scale bars proportional to the substitution rates; support values are Bayesian Posterior/ML probability support; support values below 0.5 are not shown; species names in red font indicate fish species that are further identified by morphological taxonomy; and results of DNA-based classification from ASAP and BPP are also integrated into the *COI* phylogenetic tress, as indicated by solid black/red circles.

Both the Bayesian inference and maximum likelihood calculations and analyses generated phylogenetic trees with similar topologies. The phylogenetic trees suggested that all Antarctic fish species developed different clusters, and most species then stayed with their conspecifics in monophyletic clades with high bootstrap values. However, a haplotype sequence identified as *Cygnodraco mawsoni* Waite, 1916 was nested within Artedidraconidae and Channichthyidae with a high bootstrap support value, while separating from its sibling members within Bathydraconidae (Figure 2).

In summary, our study identified 24 species belonging to 19 genera and 11 families, namely, Paralepididae (2 species, 2 specimens), Macrouridae (2 species, 34 specimens), Muraenolepididae (2 species, 2 specimens), Myctophidae (4 species, 11 specimens), Bathylagidae (1 species, 3 specimens), Artedidraconidae (2 species, 4 specimens), Bathydraconidae (4 species, 32 specimens), Channichthyidae (2 species, 2 specimens), Nototheniidae (2 species, 5 specimens), Zoarcidae (2 species, 2 specimens) and Liparidae (1 species, 1 specimens), with Macrouridae being the most abundant family in terms of specimen number and Bathydraconidae and Myctophidae being the most diverse families in terms of species richness (Figure 2, Table 1). The main groups across different taxonomic levels revealed by both conventional and molecular methods were highly similar.

### 3.3. Genetic Divergence Pattern and Phylogenetic Analysis

As expected, a hierarchical increase in the average K2P genetic distance with elevating taxonomic levels (from 0.63% to 3.98%) was recorded (Table 3). At the species level, the minimum divergence was found between *A. shackletoni* and *A. skottsbergi* (2.35%), and the maximum was detected between *Bathydraco antarcticus* Günther, 1878 and *N. microcephalus* (30.65%); at the genus level, the minimum divergence was found between *Artedidraco* Lönnberg, 1905 and *Pogonophryne* (2.64%), and the maximum was detected between *Bathylagus* and *Notomuraenobathys* Balushkin & Prirodina, 2010 (29.75%); and at the family level, the minimum divergence was found between Artedidraconidae and Bathydraconidae (9.13%), and the maximum was detected between Bathylagidae and Muraenolepididae (29.75%).

The intraspecific K2P distances as mean values ranged from 0% to 3.29% across all species, with a mean value of 0.63%, while the minimum interspecific K2P distances, as mentioned above, ranged from 2.35% to 23.86%, with a mean value of 8.94% (Table 4). The maximum intraspecific distances of all species were less than 2%, except for *B. antarcticus*, *Lycodichthys antarcticus* Pappenheim, 1911 and *N. coatsorum*, whose intraspecific distances were 2.09%, 2.18% and 3.29%, respectively. In particular, the maximum intraspecific distance of *B. antarcticus* and *N. coatsorum* even exceeded the minimum interspecific distance of some other Antarctic fish species. In this regard, although no overlap existed between intraspecific variability and interspecific variability for most fish species, a clear DNA barcoding gap cannot be applied for all taxonomic units.

## 4. Discussion

### 4.1. DNA Barcoding Demonstrated to Be an Effective Way to Discriminate Antarctic Fish Species

As the largest vertebrate taxa, fish show tremendous variabilities in colour pattern, scale size, body shape as well as fin ray type and number [52,53] and may experience drastic phenotypic changes during different developmental stages [54,55]. In addition, morphological characteristics are often of little value for species delineation due to the considerable interspecific overlaps or intraspecific invariants found in many cases [53]. Therefore, fish identification could be a challenge for taxonomists in some complicated cases, even for well-trained and highly experienced experts. Since the initiation of biological identification [56], taking the mitochondrial *COI* gene fragment as a standard DNA barcode has been proven to be a powerful approach to discriminate and identify species in various taxa. Previous studies have demonstrated that DNA barcoding with the *COI* gene can effectively discriminate and identify most Antarctic fish species in the Southern Ocean, for example, 87.5% morphological species of Southern Ocean fishes can be well resolved by *COI* barcoding [26]. In our study, although only 72.45% morphological identification results were fully supported by DNA barcoding analyses, the consistency between traditional species delimitation and molecular approach is quite close when taking our inexperienced classification skills into account. A key prerequisite for such accurate and effective species delineation is a valid barcoding gap between interspecific genetic distance and intraspecific genetic distance [57].

In this study, the validity of a barcoding gap was initially supported by ASAP but failed to be further verified by genetic divergence analysis, which was attributed to the overlap between maximum intraspecific distance of *B. antarcticus* and *N. coatsorum* and minimum interspecific distance of some other species. Previous DNA barcoding analysis on Antarctic fishes found that cryptic species might exist in genus *Bathylagus* [16] and *N. coatsorum* [26] due to their unusual high intraspecific divergent level. Although our study cannot fully prove their assumption due to insufficient samples, the fact that ASAP divided *N. coatsorum* and *B. antarcticus* into two taxonomic units, respectively, raised our attention. Meanwhile, the maximum and mean intraspecific distances of the two “species” were also greatest among all and exceeded the general intraspecific level. Therefore, there was no doubt that our finding was another important indicative proof for the existence of these potential cryptic species and thus further supported viewpoints proposed by previous studies. In addition, it was also noteworthy that the efficacy of DNA barcoding as an important taxonomic method for Antarctic fishes has not been compromised by the lack of a clear DNA barcoding gap, rather, it has been further strengthened by the indication of candidate cryptic species.

Furthermore, almost all mitochondrial *COI* barcode sequences, including those affiliated with potential cryptic species, hit against the vouchered reference well with almost unanimous (99–100%) consensus strength, including a sequence assigned to *N. microcephalus*, whose reference length was 153 bp shorter. The species identity of the sequence was finally confirmed by checking the key morphological diagnoses. Thus, a combined approach integrating both morphological taxonomy and molecular taxonomy is highly recommended, particularly for Antarctic fishes, whose reference sequences might be limited or even unavailable in public databases such as GenBank [31]. In spite of the reference sequence problems we encountered, all these results can establish solid confidence for the validity and reliability of our Antarctic fish taxonomy.

### 4.2. Demersal Fish Communities of the Cosmonaut Sea

To the best of our knowledge, there has been no systematic description of demersal fish diversity and communities in the Cosmonaut Sea that integrates both morphological taxonomy and molecular taxonomy so far, therefore, this study provided novel insights into demersal fish diversity in the Cosmonaut Sea. As revealed by our DNA barcoding results, the demersal fish fauna of the Cosmonaut Sea was characterised by typical demersal species inhabiting the shelf and upper slope around the Antarctic continent, and the most species-abundant fish groups were the notothenioids, including artedidraconids, bathydraconids, channichthyids and nototheniids, followed by myctophids, muraenolepidids, zoarcids, macrourids, liparids, bathylagids and paralepidids. Non-notothenoids were either typical deep-sea groups or representative mesopelagic members. Although many Antarctic fish species have a circumpolar distribution, local fish communities of different regions may still vary regionally when latitude and location change [12]. For example, in the ice-free Sub-Antarctic island shelf areas, predominant members of local demersal fish communities include the channichthyids *Champsocephalus gunnari* Lönnberg, 1905 and *Chaenocephalus aceratus* (Lönnberg, 1906), the nototheniids *Lepidonotothen* Balushkin, 1976 spp. and *Notothenia* Richardson, 1844 spp. as well as *Gobionotothen gibberifrons* (Lönnberg, 1905) and *Patagonotothen guntheri* (Norman, 1937) [27]. In the seasonal sea ice zones located in higher latitudes, such as the neritic waters of the Antarctic Peninsula, benthic fish fauna was most likely to be bathydraconids *Parachaenichthys charcoti* (Vaillant, 1906), channichthyids *Pagetopsis macropterus* (Boulenger, 1907) and *Pseudochaenichthys georgianus* Norman, 1937, nototheniids *Lepidonotothen* spp. and *Notothenia* spp. and *Harpagifer antarcticus* Nybelin, 1947 (Harpagiferidae) [6]; in the high-latitude Antarctic shelf regions, such as the Dumont d’Urville Sea and Ross Sea, demersal fish communities consisted of artedidraconids, bathydraconids, channichthyids and nototheniids, mainly *Pleuragramma antarcticum* Boulenger, 1902 and *Trematomus* Boulenger, 1902 spp. Other non-notothenioids were deep-sea fishes such as paralepidids, macrourids, muraenolepidids, myctophids, bathylagids, zoarcids and liparids [26,58]. In this regard, the demersal fish ichthyofauna inhabiting Cosmonaut Sea match the typical pattern at high latitudes of the Southern Ocean more than that of Sub-Antarctic island shelf areas and seasonal sea ice zones of higher latitudes, which was consistent with our anticipation as well. When compared with demersal fish communities of other Eastern Antarctica areas at high latitudes, such as Prydz Bay, which is dominated by Artedidraconidae, Bathydraconidae, Channichthyidae, Nototheniidae (*Trematomus* spp. and *P. antarcticum*), Zoarcidae and Rajidae [12], almost all groups were also found in the Cosmonaut Sea. In addition, additional members of Paralepididae, Macrouridae, Muraenolepididae, Myctophidae, Bathylagidae and Liparidae, most of which were more likely to be mesopelagic fish, were identified in our study. However, it was noteworthy that skates, a dominant chondrichthyan group in Antarctic waters, were absent in our fish samples because skates in the Southern Ocean were more likely to be captured as the bycatch of toothfish longline vessels [26] and less likely to be harvested by bottom trawling. Despite the absence of skates, the benthic ichthyofauna of the Cosmonaut Sea still resembles the typical pattern of fish communities in high-latitude Antarctic waters to a very large extent.

### 4.3. Comparison of Our Results and Fish Occurrence Records in the Cosmonaut Sea

For the absence of seventeen fish species previously recorded in the book chapter, there were several possible explanations. First, the trawl stations were not capable of covering all sections of the Cosmonaut Sea. For example, *Pachycara* Zugmayer, 1911 spp. was mainly distributed in neritic zones of Lützow-Holm Bay [17], which was beyond the targeted exploration area of the 37th and 38th CHINARE cruises. Second, the trawling depths of all stations were not successive, with a missing stratum of approximately 300–800 m in particular due to the combined effects of sea ice cover, bottom topography, substrate pattern and other random factors on trawling station setting. However, fish species such as *P. permitini* Andriashev, 1967, *Gerlachea australis* Dollo, 1900 and *Cryodraco* Dollo, 1900 spp. were recorded inhabiting this depth range only [59], which may have resulted in the failure to capture these species in our hauls. Last, as previously mentioned, the main fishing gear we used was bottom trawl. Although trawling nets may unintentionally harvest mesopelagic fish such as *Electrona antarctica* (Günther, 1878) and *Gymnoscopelus* Günther, 1873 sp., their target groups were mainly demersal fishes; thus, they can hardly, though not impossibly, capture pelagic fish such as *P. antarcticum*. For the demersal fish, not all members were still target groups. For instance, skates and toothfish were more likely to be caught by bottom longlines [26,60] than any other fishing gear, which might be the reason why we failed to observe these typical demersal fish in our catch. Even so, our DNA barcoding analysis of fish samples has substantially expanded current knowledge regarding fish biodiversity in the Cosmonaut Sea. Meanwhile, the novel distribution records for the nine species supported by molecular taxonomy indicated that the Cosmonaut Sea was poorly explored even in comparison with other seas in the Southern Ocean. In the future, more sampling efforts, including but not limited to the use of various fishing gear and eDNA methods, are encouraged to fully cover the geographic range and depth stratum of the Cosmonaut Sea to enhance our understanding of endemic fish biodiversity, within which DNA barcoding can play an important role in either molecular taxonomy or the establishment of a local reference database in eDNA analyses by contributing 12S rDNA and 16S rDNA barcodes that are urgently needed [31].

## 5. Conclusions

By implementing DNA barcoding analysis on fish samples collected by trawling during 37th and 38th CHINARE cruises, we provided novel insights into the demersal fish diversity of the Cosmonaut Sea. Twenty-four species representing 19 genera and 11 families, namely, Artedidraconidae, Bathydraconidae, Bathylagidae, Channichthyidae, Liparidae, Macrouridae, Muraenolepididae, Myctophidae, Nototheniidae, Paralepididae and Zoarcidae were characterised, which was highly identical to historical records. Nevertheless, skates and some other fish species that were previously observed were absent in our catches, which was due to the biased station setting, incomplete coverage of specific depths and selectivity of fishing gear. Furthermore, a novel description of the occurrence of nine fish species was added by our sampling effort and DNA taxonomy, implying that our knowledge regarding fish diversity in the Cosmonaut Sea has been sparse. In general, the demersal fish diversity of the Cosmonaut Sea was consistent with the general pattern of ichthyofauna in the high-latitude Antarctic seas. However, various sampling strategies that cover all geographic sections and depth strata are still encouraged to further improve our understanding of local fish communities, within which DNA barcoding can play a significant role in either molecular taxonomy or the establishment of a dedicated local reference database for eDNA metabarcoding analyses.

## Figures and Tables

**Table 1:** Summary of fish specimens and species identities using both morphological and molecular taxonomies.

Sample ID	Cruise	Trawling Station	Longitude (°/E)	Latitude (°/S)	Trawling Depth (m)	Trawling Duration (min)	Morphological Taxonomy	Molecular Taxonomy
1	CHINARE-37	C2P-13	33.7361	67.3241	1270	15	*Macrourus whitsoni* (Regan, 1913)	*Macrourus whitsoni* (Regan, 1913)
2	CHINARE-37	C2P-13	33.7361	67.3241	1270	15	*Macrourus whitsoni* (Regan, 1913)	*Macrourus whitsoni* (Regan, 1913)
3	CHINARE-37	C2P-13	33.7361	67.3241	1270	15	*Macrourus whitsoni* (Regan, 1913)	*Macrourus whitsoni* (Regan, 1913)
4	CHINARE-37	C2P-13	33.7361	67.3241	1270	15	*Macrourus whitsoni* (Regan, 1913)	*Macrourus whitsoni* (Regan, 1913)
5	CHINARE-37	C2P-13	33.7361	67.3241	1270	15	*Macrourus whitsoni* (Regan, 1913)	*Macrourus whitsoni* (Regan, 1913)
6	CHINARE-37	C2P-13	33.7361	67.3241	1270	15	*Electrona antarctica* (Günther, 1878)	*Electrona antarctica* (Günther, 1878)
7	CHINARE-37	C2P-13	33.7361	67.3241	1270	15	*Electrona antarctica* (Günther, 1878)	*Electrona antarctica* (Günther, 1878)
8	CHINARE-37	C2P-13	33.7361	67.3241	1270	15	*Bathylagus antarcticus* Günther, 1878	*Bathylagus antarcticus* Günther, 1878
9	CHINARE-37	C4-12	44.3405	67.1106	986	15	*Bathylagus antarcticus* Günther, 1878	*Bathylagus antarcticus* Günther, 1878
10	CHINARE-37	C4-11	45.0542	66.4743	2085	15	*Electrona antarctica* (Günther, 1878)	*Electrona antarctica* (Günther, 1878)
11	CHINARE-37	C4-11	45.0542	66.4743	2085	15	*Electrona antarctica* (Günther, 1878)	*Electrona antarctica* (Günther, 1878)
12	CHINARE-37	C4-11	45.0542	66.4743	2085	15	*Gymnoscopelus opisthopterus* Fraser-Brunner, 1949	*Gymnoscopelus opisthopterus* Fraser-Brunner, 1949
13	CHINARE-37	C4-11	45.0542	66.4743	2085	15	*Coryphaenoides lecointei* (Dollo, 1900)	*Coryphaenoides lecointei* (Dollo, 1900)
14	CHINARE-37	C4-11	45.0542	66.4743	2085	15	*Macrourus whitsoni* (Regan, 1913)	*Macrourus whitsoni* (Regan, 1913)
15	CHINARE-37	C4-11	45.0542	66.4743	2085	15	*Bathydraco antarcticus* Günther, 1878	*Bathydraco antarcticus* Günther, 1878
16	CHINARE-37	C4-11	45.0542	66.4743	2085	15	*Macrourus whitsoni* (Regan, 1913)	*Macrourus whitsoni* (Regan, 1913)
17	CHINARE-37	C4-11	45.0542	66.4743	2085	15	*Careproctus longipectoralis* Duhamel, 1992	*Careproctus longipectoralis* Duhamel, 1992
18	CHINARE-37	C2P-06	34.1758	65.3239	1763	15	*Macrourus whitsoni* (Regan, 1913)	*Macrourus whitsoni* (Regan, 1913)
19	CHINARE-37	C2P-06	34.1758	65.3239	1763	15	*Macrourus whitsoni* (Regan, 1913)	*Macrourus whitsoni* (Regan, 1913)
20	CHINARE-37	C2P-06	34.1758	65.3239	1763	15	*Macrourus whitsoni* (Regan, 1913)	*Macrourus whitsoni* (Regan, 1913)
21	CHINARE-37	C2P-06	34.1758	65.3239	1763	15	*Macrourus whitsoni* (Regan, 1913)	*Macrourus whitsoni* (Regan, 1913)
22	CHINARE-37	C2P-06	34.1758	65.3239	1763	15	*Macrourus whitsoni* (Regan, 1913)	*Macrourus whitsoni* (Regan, 1913)
23	CHINARE-37	C2P-06	34.1758	65.3239	1763	15	*Macrourus whitsoni* (Regan, 1913)	*Macrourus whitsoni* (Regan, 1913)
24	CHINARE-37	C2P-06	34.1758	65.3239	1763	15	*Macrourus whitsoni* (Regan, 1913)	*Macrourus whitsoni* (Regan, 1913)
25	CHINARE-37	C2P-06	34.1758	65.3239	1763	15	*Macrourus whitsoni* (Regan, 1913)	*Macrourus whitsoni* (Regan, 1913)
26	CHINARE-37	C4-11	45.0542	66.4743	2085	15	*Macrourus whitsoni* (Regan, 1913)	*Macrourus whitsoni* (Regan, 1913)
27	CHINARE-37	C4-11	45.0542	66.4743	2085	15	*Macrourus whitsoni* (Regan, 1913)	*Macrourus whitsoni* (Regan, 1913)
28	CHINARE-37	C56-09	52.5742	65.5688	1940	15	*Macrourus whitsoni* (Regan, 1913)	*Macrourus whitsoni* (Regan, 1913)
29	CHINARE-37	C56-09	52.5742	65.5688	1940	15	*Gymnoscopelus opisthopterus* Fraser-Brunner, 1949	*Gymnoscopelus opisthopterus* Fraser-Brunner, 1949
30	CHINARE-38	C2P-06	34.1713	65.1852	1492	15	*Muraenolepis orangiensis* Vaillant, 1888	*Muraenolepis orangiensis* Vaillant, 1888
31	CHINARE-38	C2P-06	34.1713	65.1852	1492	15	*Bathylagus* Günther, 1878 sp.	*Bathylagus antarcticus* Günther, 1878 *
32	CHINARE-38	C2P-06	34.1713	65.1852	1492	15	*Coryphaenoides* Gunnerus, 1765 sp.1	*Macrourus whitsoni* (Regan, 1913) *
33	CHINARE-38	C6P-08	52.4918	65.6517	270	15	*Lycodichthys antarcticus* Pappenheim, 1911	*Lycodichthys antarcticus* Pappenheim, 1911
34	CHINARE-38	C4-11	45.0173	66.6722	2159	15	*Muraenolepis microps* Lönnberg, 1905	*Notomuraenobathys* *microcephalus* (Norman, 1937) *
35	CHINARE-38	C4-11	45.0173	66.6722	2159	15	*Coryphaenoides* Gunnerus, 1765 sp.2	*Macrourus whitsoni* (Regan, 1913) *
36	CHINARE-38	C4-11	45.0173	66.6722	2159	15	*Bathydraco joannae* DeWitt, 1985	*Bathydraco antarcticus* Günther, 1878 *
37	CHINARE-38	C4-11	45.0173	66.6722	2159	15	*Bathydraco joannae* DeWitt, 1985	*Bathydraco antarcticus* Günther, 1878 *
38	CHINARE-38	C4-11	45.0173	66.6722	2159	15	*Bathydraco joannae* DeWitt, 1985	*Bathydraco antarcticus* Günther, 1878 *
39	CHINARE-38	C4-11	45.0173	66.6722	2159	15	*Bathydraco antarcticus* Günther, 1878	*Bathydraco antarcticus* Günther, 1878
40	CHINARE-38	C5P-05	47.4897	64.6615	250	30	*Chionobathyscus dewitti* Andriashev & Neelov, 1978	*Chionobathyscus dewitti* Andriashev & Neelov, 1978
41	CHINARE-38	C4-11	45.0173	66.6722	2159	15	*Macrourus whitsoni* (Regan, 1913)	*Macrourus whitsoni* (Regan, 1913)
42	CHINARE-38	C4-11	45.0173	66.6722	2159	15	*Macrourus whitsoni* (Regan, 1913)	*Macrourus whitsoni* (Regan, 1913)
43	CHINARE-38	C4-11	45.0173	66.6722	2159	15	*Macrourus whitsoni* (Regan, 1913)	*Macrourus whitsoni* (Regan, 1913)
44	CHINARE-38	C4-11	45.0173	66.6722	2159	15	*Lycenchelys antarctica* Regan, 1913	*Lycenchelys aratrirostris* Andriashev & Permitin, 1968 *
45	CHINARE-38	C4-12	44.9958	67.0017	1679	15	*Notolepis* Dollo, 1908 sp.1	*Notolepis coatsorum* Dollo, 1908 *
46	CHINARE-38	C4-12	44.9958	67.0017	1679	15	*Notolepis* Dollo, 1908 sp.2	*Notolepis coatsorum* Dollo, 1908 *
47	CHINARE-38	C4-12	44.9958	67.0017	1679	15	*Gymnoscopelus braueri* (Lönnberg, 1905)	*Gymnoscopelus braueri* (Lönnberg, 1905)
48	CHINARE-38	C4-12	44.9958	67.0017	1679	15	*Electrona antarctica* (Günther, 1878)	*Electrona antarctica* (Günther, 1878)
49	CHINARE-38	CA1-10	48.7242	66.3123	1004	15	*Bathydraco antarcticus* Günther, 1878	*Bathydraco antarcticus* Günther, 1878
50	CHINARE-38	CA1-10	48.7242	66.3123	1004	15	*Macrourus whitsoni* (Regan, 1913)	*Macrourus whitsoni* (Regan, 1913)
51	CHINARE-38	CA1-10	48.7242	66.3123	1004	15	*Macrourus whitsoni* (Regan, 1913)	*Macrourus whitsoni* (Regan, 1913)
52	CHINARE-38	CA1-10	48.7242	66.3123	1004	15	*Macrourus whitsoni* (Regan, 1913)	*Macrourus whitsoni* (Regan, 1913)
53	CHINARE-38	CA1-10	48.7242	66.3123	1004	15	*Macrourus whitsoni* (Regan, 1913)	*Macrourus whitsoni* (Regan, 1913)
54	CHINARE-38	CA1-10	48.7242	66.3123	1004	15	*Macrourus whitsoni* (Regan, 1913)	*Macrourus whitsoni* (Regan, 1913)
55	CHINARE-38	CA1-10	48.7242	66.3123	1004	15	*Macrourus whitsoni* (Regan, 1913)	*Macrourus whitsoni* (Regan, 1913)
56	CHINARE-38	CA2-09	51.2150	65.9750	229	15	*Chionodraco hamatus* (Lönnberg, 1905)	*Chionodraco hamatus* (Lönnberg, 1905)
57	CHINARE-38	CA2-09	51.2150	65.9750	229	15	*Trematomus pennellii* Regan, 1914	*Trematomus pennellii* Regan, 1914
58	CHINARE-38	CA2-09	51.2150	65.9750	229	15	*Artedidraco shackletoni* Waite, 1911	*Artedidraco shackletoni* Waite, 1911
59	CHINARE-38	CA2-09	51.2150	65.9750	229	15	*Cygnodraco mawsoni* Waite, 1916	*Cygnodraco mawsoni* Waite, 1916
60	CHINARE-38	CA2-09	51.2150	65.9750	229	15	*Prionodraco evansii* Regan, 1914	*Prionodraco evansii* Regan, 1914)
61	CHINARE-38	CA2-09	51.2150	65.9750	229	15	*Prionodraco evansii* Regan, 1914	*Prionodraco evansii* Regan, 1914)
62	CHINARE-38	CA2-09	51.2150	65.9750	229	15	*Prionodraco evansii* Regan, 1914	*Prionodraco evansii* Regan, 1914)
63	CHINARE-38	CA2-09	51.2150	65.9750	229	15	*Prionodraco evansii* Regan, 1914	*Prionodraco evansii* Regan, 1914
64	CHINARE-38	CA1-09	49.0147	65.9546	1872	15	*Macrourus whitsoni* (Regan, 1913)	*Macrourus whitsoni* (Regan, 1914)
65	CHINARE-38	CA1-09	49.0147	65.9546	1872	15	*Macrourus whitsoni* (Regan, 1913)	*Macrourus whitsoni* (Regan, 1913)
66	CHINARE-38	CA1-09	49.0147	65.9546	1872	15	*Macrourus whitsoni* (Regan, 1913)	*Macrourus whitsoni* (Regan, 1913)
67	CHINARE-38	CA1-09	49.0147	65.9546	1872	15	*Macrourus whitsoni* (Regan, 1913)	*Macrourus whitsoni* (Regan, 1913)
68	CHINARE-38	CA1-09	49.0147	65.9546	1872	15	*Electrona antarctica* (Günther, 1878)	*Electrona antarctica* (Günther, 1878)
69	CHINARE-38	CA3-08	53.8283	65.5457	240	15	*Racovitzia glacialis* Dollo, 1900	*Racovitzia glacialis* Dollo, 1900
70	CHINARE-38	CA3-08	53.8283	65.5457	240	5	*Racovitzia glacialis* Dollo, 1900	*Racovitzia glacialis* Dollo, 1900
71	CHINARE-38	CA3-08	53.8283	65.5457	240	5	*Pogonophryne* Regan, 1914 sp.1	*Pogonophryne scotti* Regan, 1914 *
72	CHINARE-38	CA3-08	53.8283	65.5457	240	5	*Pogonophryne* Regan, 1914 sp.2	*Pogonophryne scotti* Regan, 1914 *
73	CHINARE-38	CA3-08	53.8283	65.5457	240	5	*Trematomus scotti* (Boulenger, 1907)	*Trematomus scotti* (Boulenger, 1907)
74	CHINARE-38	CA3-08	53.8283	65.5457	240	5	*Trematomus scotti* (Boulenger, 1907)	*Trematomus scotti* (Boulenger, 1907)
75	CHINARE-38	CA3-08	53.8283	65.5457	240	5	*Trematomus scotti* (Boulenger, 1907)	*Trematomus scotti* (Boulenger, 1907)
76	CHINARE-38	CA3-08	53.8283	65.5457	240	5	*Trematomus scotti* Boulenger, 1907	*Artedidraco skottsbergi* Lönnberg, 1905 *
77	CHINARE-38	CA3-08	53.8283	65.5457	240	5	*Trematomus scotti* Boulenger, 1907	*Trematomus scotti* (Boulenger, 1907)
78	CHINARE-38	CA3-08	53.8283	65.5457	240	5	*Prionodraco evansii* Regan, 1914	*Prionodraco evansii* Regan, 1914
79	CHINARE-38	CA3-08	53.8283	65.5457	240	5	*Prionodraco evansii* Regan, 1914	*Prionodraco evansii* Regan, 1914
80	CHINARE-38	CA3-08	53.8283	65.5457	240	5	*Prionodraco evansii* Regan, 1914	*Prionodraco evansii* Regan, 1914
81	CHINARE-38	CA3-08	53.8283	65.5457	240	5	*Prionodraco evansii* Regan, 1914	*Prionodraco evansii* Regan, 1914
82	CHINARE-38	CA3-08	53.8283	65.5457	240	5	*Prionodraco evansii* Regan, 1914	*Prionodraco evansii* Regan, 1914
83	CHINARE-38	CA3-08	53.8283	65.5457	240	5	*Prionodraco evansii* Regan, 1914	*Prionodraco evansii* Regan, 1914
84	CHINARE-38	CA3-08	53.8283	65.5457	240	5	*Prionodraco evansii* Regan, 1914	*Prionodraco evansii* Regan, 1914
85	CHINARE-38	CA3-08	53.8283	65.5457	240	5	*Trematomus scotti* (Boulenger, 1907)	*Prionodraco evansii* Regan, 1914 *
86	CHINARE-38	CA3-08	53.8283	65.5457	240	5	*Trematomus scotti* (Boulenger, 1907)	*Prionodraco evansii* Regan, 1914 *
87	CHINARE-38	CA3-08	53.8283	65.5457	240	5	*Trematomus scotti* (Boulenger, 1907)	*Prionodraco evansii* Regan, 1914 *
88	CHINARE-38	CA3-08	53.8283	65.5457	240	5	*Trematomus scotti* (Boulenger, 1907)	*Prionodraco evansii* (Regan, 1914 *
89	CHINARE-38	CA3-08	53.8283	65.5457	240	5	*Trematomus scotti* (Boulenger, 1907)	*Prionodraco evansii* Regan, 1914 *
90	CHINARE-38	CA3-08	53.8283	65.5457	240	5	*Trematomus scotti* (Boulenger, 1907)	*Prionodraco evansii* Regan, 1914 *
91	CHINARE-38	CA3-08	53.8283	65.5457	240	5	*Trematomus scotti* (Boulenger, 1907)	*Prionodraco evansii* Regan, 1914 *
92	CHINARE-38	CA3-08	53.8283	65.5457	240	5	*Trematomus scotti* (Boulenger, 1907)	*Prionodraco evansii* Regan, 1914 *
93	CHINARE-38	CA3-08	53.8283	65.5457	240	5	*Trematomus scotti* (Boulenger, 1907)	*Prionodraco evansii* Regan, 1914 *
94	CHINARE-38	CA3-08	53.8283	65.5457	240	5	*Trematomus scotti* (Boulenger, 1907)	*Prionodraco evansii* Regan, 1914 *
95	CHINARE-38	CA3-08	53.8283	65.5457	240	5	*Trematomus scotti* (Boulenger, 1907)	*Prionodraco evansii* Regan, 1914 *
96	CHINARE-38	CA3-08	53.8283	65.5457	240	5	*Trematomus scotti* (Boulenger, 1907)	*Prionodraco evansii* Regan, 1914 *
97	CHINARE-38	C7-11	59.9998	66.6647	854	15	*Electrona antarctica* (Günther, 1878)	*Electrona antarctica* (Günther, 1878)
98	CHINARE-38	C7P-07	59.9968	65.3302	250	15	*Gymnoscopelus braueri* (Lönnberg, 1905)	*Gymnoscopelus nicholsi* (Gilbert, 1911) *

Note: * indicates initial ambiguous or incorrectly identified species that were identified by molecular taxonomy.

**Table 2:** Comparison of historical records and CHINARE results of fish occurrence in the Cosmonaut Sea.

Historical Records		CHINARE Results	
Family	Species	Family	Species
Rajidae	*Amblyraja georgiana* (Norman, 1938)		
	*Bathyraja eatonii* (Günther, 1876)		
	*Bathyraja maccaini* Springer, 1971		
Paralepididae	*Notolepis coatsorum*	Paralepididae	*Notolepis coatsorum*
Macrouridae	*Macrourus caml* McMillan, Iwamoto, Stewart & Smith, 2012	Macrouridae	*Coryphaenoides lecointei* *Macrourus whitsoni*
	*Macrourus whitsoni*		
	*Antimora rostrata* (Günther, 1878)		
Muraenolepididae	*Muraenolepis marmorata* Günther, 1880	Muraenolepididae	*Muraenolepis orangiensis*
			*Notomuraenobathys* *microcephalus*
Myctophidae	*Electrona antarctica* *Gymnoscopelus braueri* *Gymnoscopelus nicholsi*	Myctophidae	*Electrona antarctica*
			*Gymnoscopelus braueri*
			*Gymnoscopelus nicholsi*
			*Gymnoscopelus opisthopterus*
Bathylagidae	*Bathylagus* sp.	Bathylagidae	*Bathylagus antarcticus*
Artedidraconidae	*Artedidraco* spp.	Artedidraconidae	*Artedidraco shackletoni*
	*Pogonophryne permitini* Andriashev, 1967		*Pogonophryne scotti*
Bathydraconidae	*Cygnodraco mawsoni*	Bathydraconidae	*Cygnodraco mawsoni*
	*Gerlachea australis* Dollo, 1900		*Bathydraco antarcticus*
	*Prionodraco evansii*		*Prionodraco evansii*
	*Gymnodraco acuticeps* Boulenger, 1902		*Racovitzia glacialis*
Channichthyidae	*Chaenodraco wilsoni* Regan, 1914	Channichthyidae	*Chionodraco hamatus*
	*Chionobathyscus dewitti*		*Chionobathyscus dewitti*
	*Cryodraco* spp.		
Nototheniidae	*Lepidonotothen squamifrons* (Günther, 1880)	Nototheniidae	
	*Dissostichus mawsoni* Norman, 1937		
	*Pleuragramma antarctica*		
	*Trematomus brachysoma* Pappenheim, 1912		*Trematomus pennellii*
	*Trematomus* spp.		*Trematomus scotti*
Zoarcidae	*Lycodichthys* Pappenheim, 1911 spp.	Zoarcidae	*Lycodichthys antarcticus*
	*Pachycara* Zugmayer, 1911 spp.		*Lycenchelys aratrirostris*
Liparidae	*Paraliparis leobergi* Andriashev, 1982	Liparidae	*Careproctus longipectoralis*

**Table 3:** Details of genetic divergence (K2P percentage) across different taxonomic levels.

	Maximum Divergence (%)	Minimum Divergence (%)	Mean Divergence (%)	SE Divergence (%)
Within species	3.29	0	0.63	0.20
Within genus	7.27	0	1.68	0.33
Within family	10.91	1.14	3.98	0.56

**Table 4:** Details of interspecific and intraspecific divergence among species based on K2P distances.

Species Name	Minimum Interspecific Distance	Mean Interspecific Distance	Maximum Intraspecific Distance	Mean Intraspecific Distance
*Artedidraco shackletoni*	0.0235	0.1779	0.0015	0.0010
*Artedidraco skottsbergi*	0.0235	0.1795	0.0046	0.0031
*Bathydraco antarcticus*	0.0358	0.1810	0.0093	0.0031
*Bathylagus antarcticus*	0.2350	0.2612	0.0310	0.0209
*Careproctus longipectoralis*	0.2386	0.2679	0.0171	0.0114
*Chionobathyscus dewitti*	0.0303	0.1905	0.0061	0.0041
*Chionodraco hamatus*	0.0303	0.1885	0.0031	0.0021
*Coryphaenoides lecointei*	0.2108	0.2531	0	0
*Cygnodraco mawsoni*	0.0694	0.1816	0.0015	0.0010
*Electrona antarctica*	0.1776	0.2297	0.0031	0.0012
*Gymnoscopelus braueri*	0.0262	0.2256	0.0015	0.0010
*Gymnoscopelus nicholsi*	0.0262	0.2344	0.0077	0.0062
*Gymnoscopelus opisthopterus*	0.0568	0.2273	0.0015	0.0010
*Lycenchelys aratrirostris*	0.0306	0.2299	0.0031	0.0021
*Lycodichthys antarcticus*	0.0306	0.2313	0.0218	0.0156
*Macrourus whitsoni*	0.2108	0.2622	0.0093	0.0013
*Notomuraenobathys* *microcephalus*	0.0825	0.2665	NA	NA
*Muraenolepis orangiensis*	0.0825	0.2596	NA	NA
*Notolepis coatsorum*	0.2128	0.2486	0.0398	0.0329
*Pogonophryne scotti*	0.0256	0.1824	0.0077	0.0062
*Prionodraco evansii*	0.0508	0.1796	0.0061	0.0022
*Racovitzia glacialis*	0.0358	0.1738	0.0031	0.0021
*Trematomus pennellii*	0.1001	0.2229	0.0015	0.0010
*Trematomus scotti*	0.1001	0.2201	0.0031	0.0015

Note: NA indicates not applicable due to the absence of barcode sequence from explicitly identified species in GenBank database.

## Data Availability

Data were deposited in GenBank under the accession number PP218555-PP218652.

## Supplementary Materials

The following supporting information can be downloaded at: https://www.mdpi.com/article/10.3390/genes15060691/s1, Figure S1: Morphological photos of *Notomuraenobathys microcephalus* (Norman, 1937); Figure S2: Morphological photos of *Muraenolepis orangiensis* Vaillant, 1888; Figure S3: Morphological photos of *Electrona antarctica* (Günther, 1878); Figure S4: Morphological photos of *Gymnoscopelus braueri* (Lönnberg, 1905); Figure S5: Morphological photos of *Bathylagus antarcticus* Günther, 1878; Figure S6: Morphological photos of *Notolepis coatsorum* Dollo, 1908; Figure S7: Morphological photos of *Macrourus whitsoni* (Regan, 1913); Figure S8: Morphological photos of *Chionobathyscus dewitti* Andriashev & Neelov 1978.; Figure S9: Morphological photos of *Cygnodraco mawsoni* Waite 1916; Figure S10: Morphological photos of *Prionodraco evansii* Regan 1914.; Figure S11: Morphological photos of *Racovitzia glacialis* Dollo 1900; Figure S12: Morphological photos of *Trematomus scotti* (Boulenger 1907).
