# Supplementary material for: Biodiversity of Demersal Fish Communities in the Cosmonaut Sea Revealed by DNA Barcoding Analyses
Source: Genes (Basel). 2024 May 26;15(6):691. doi: 10.3390/genes15060691 (PMC11202926; doi:10.3390/genes15060691)
Supplement: Supplementary file 1 [file genes-15-00691-s001.zip › genes-2968645-supplementary.pdf]

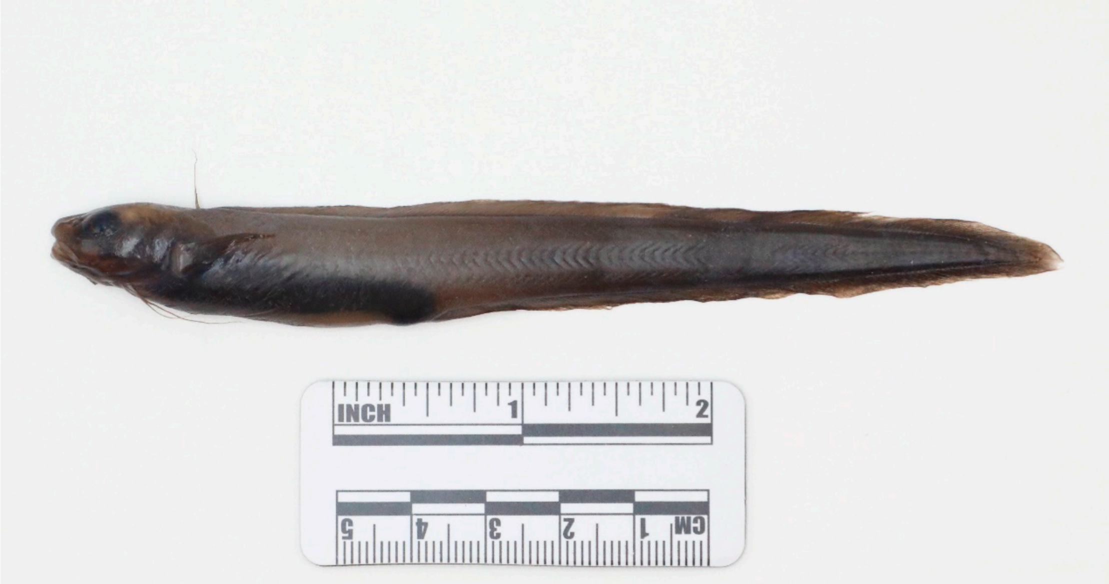

Figure S1 Morphological photos of *Notomuraenobathys microcephalus* (Norman, 1937).

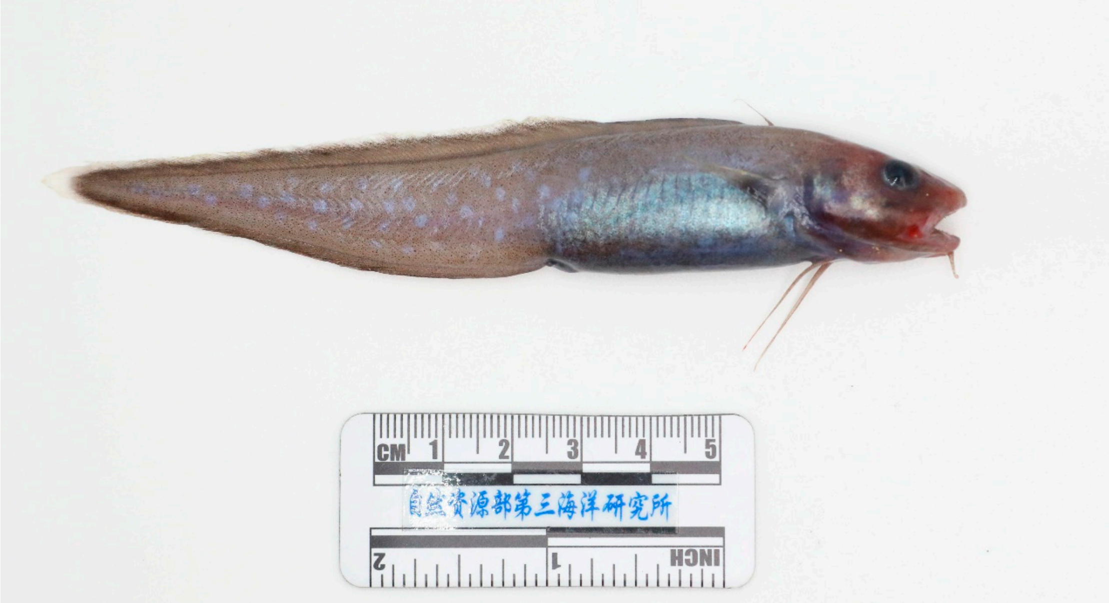

Figure S2 Morphological photos of *Muraenolepis orangiensis* Vaillant, 1888.

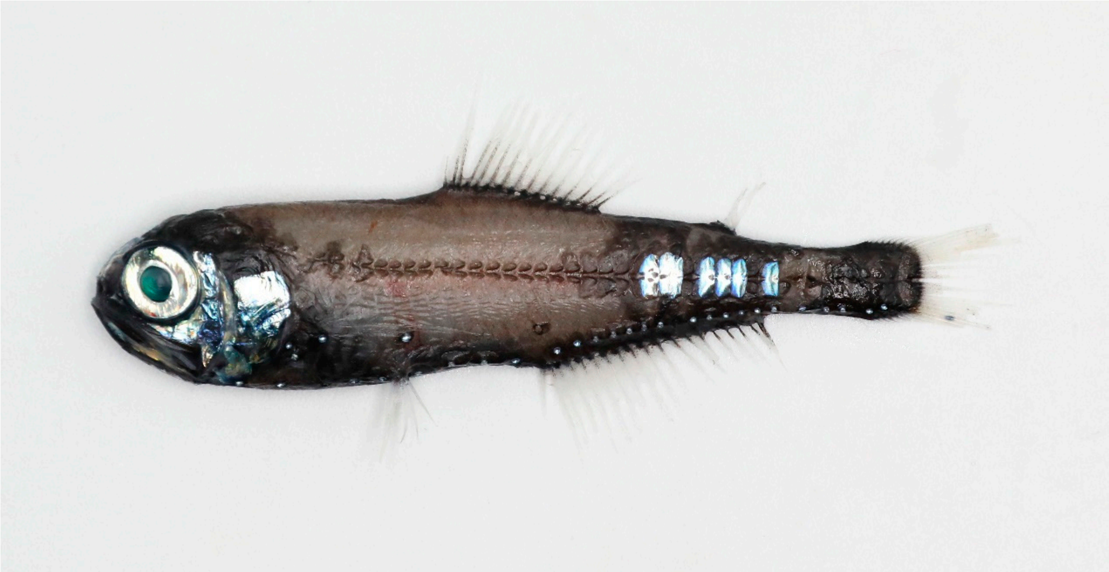

Figure S3 Morphological photos of *Electrona antarctica* (Günther, 1878).

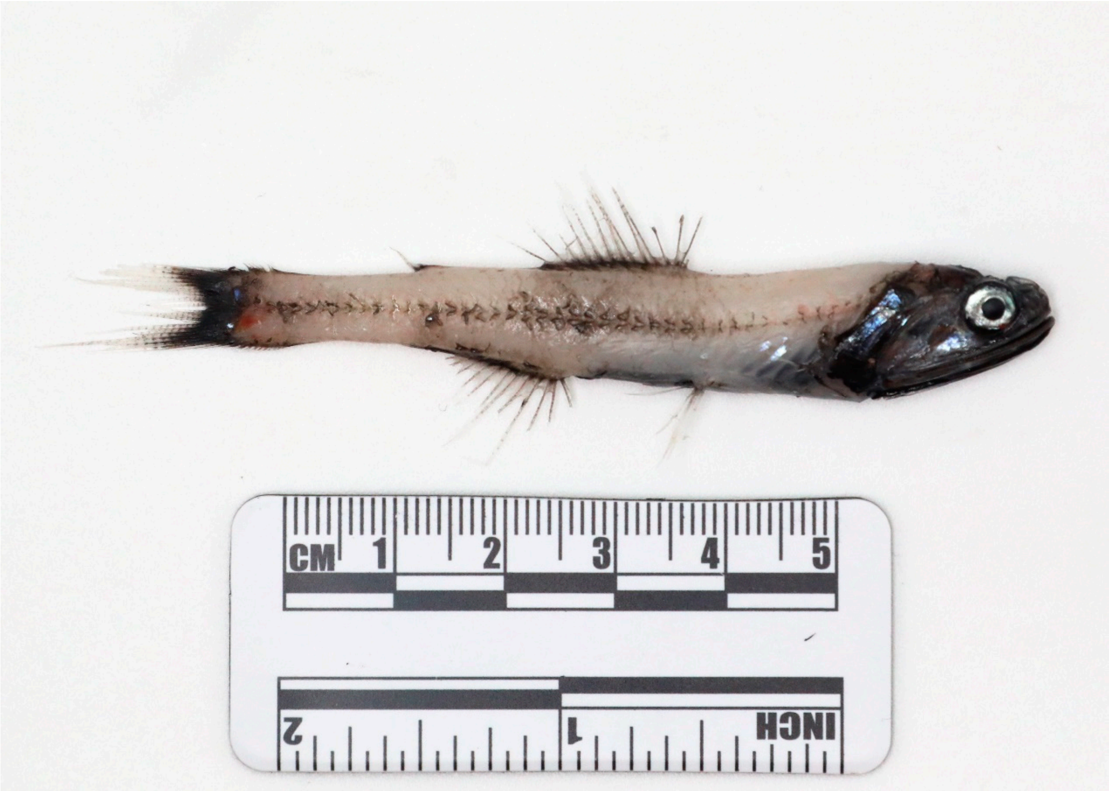

Figure S4 Morphological photos of *Gymnoscopelus braueri* (Lönnberg, 1905).

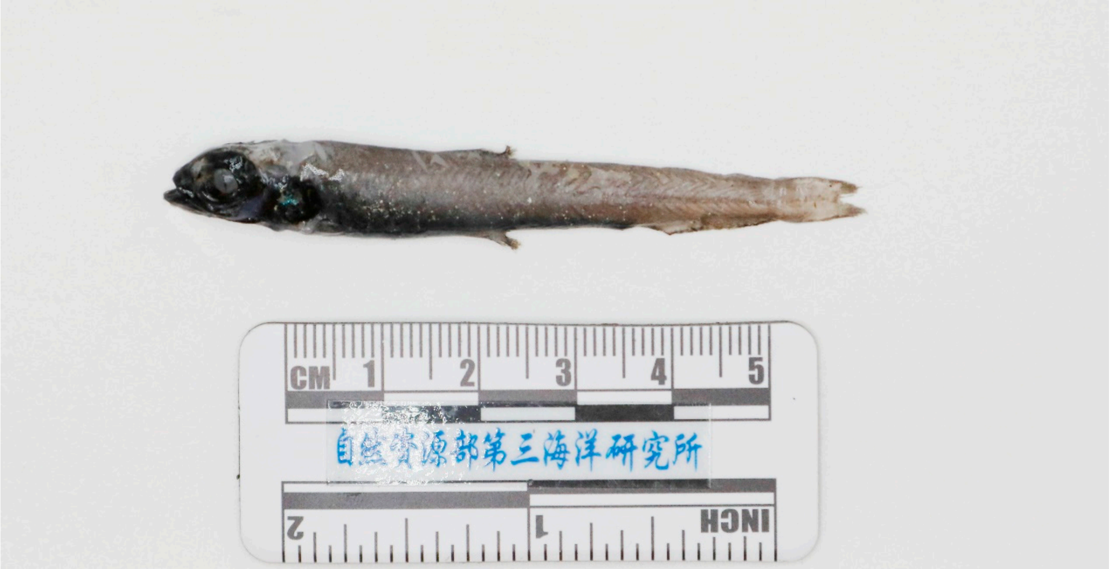

Figure S5 Morphological photos of *Bathylagus antarcticus* Günther, 1878.

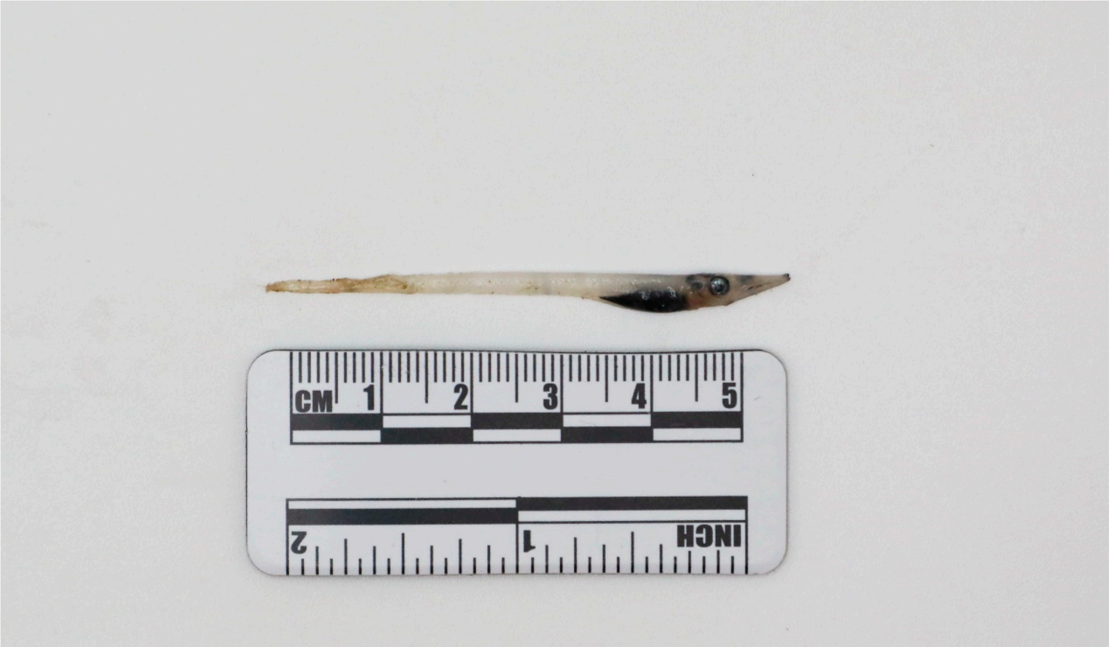

Figure S6 Morphological photos of *Notolepis coatsorum* Dollo, 1908.

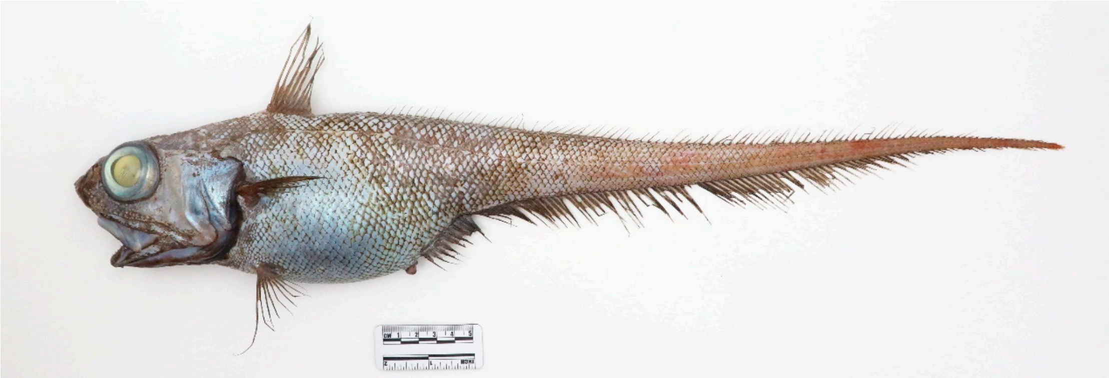

Figure S7 Morphological photos of *Macrourus whitsoni* (Regan, 1913).

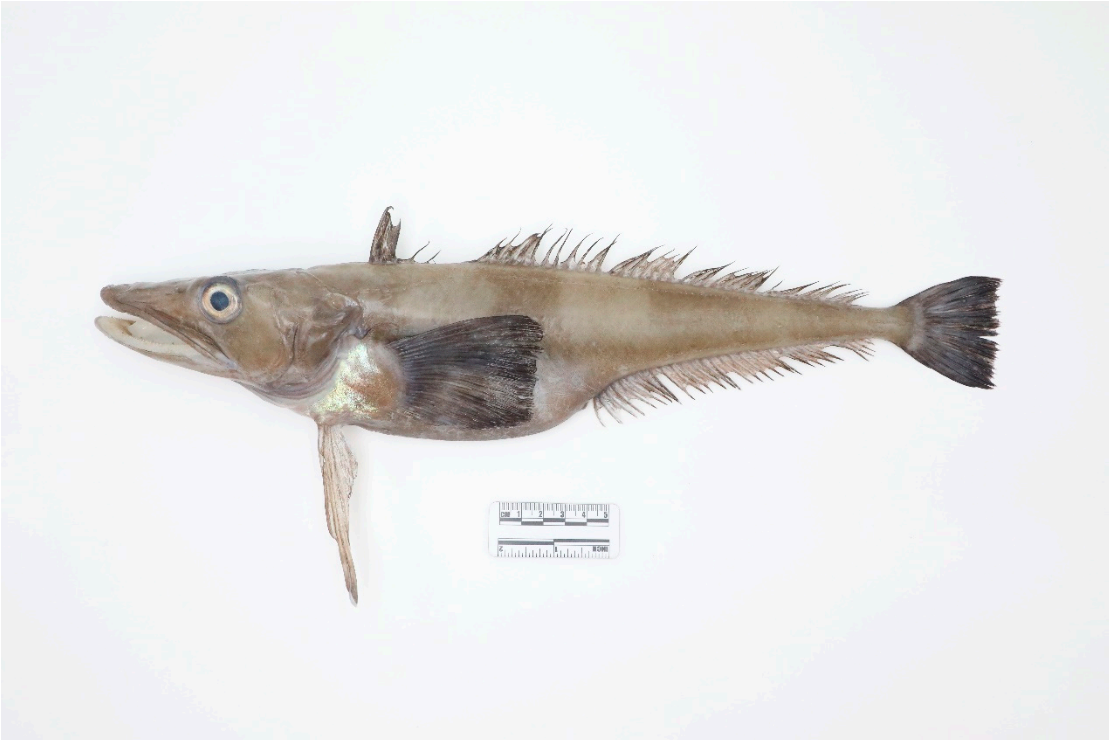

Figure S8 Morphological photos of *Chionobathyscus dewitti* Andriashev & Neelov 1978.

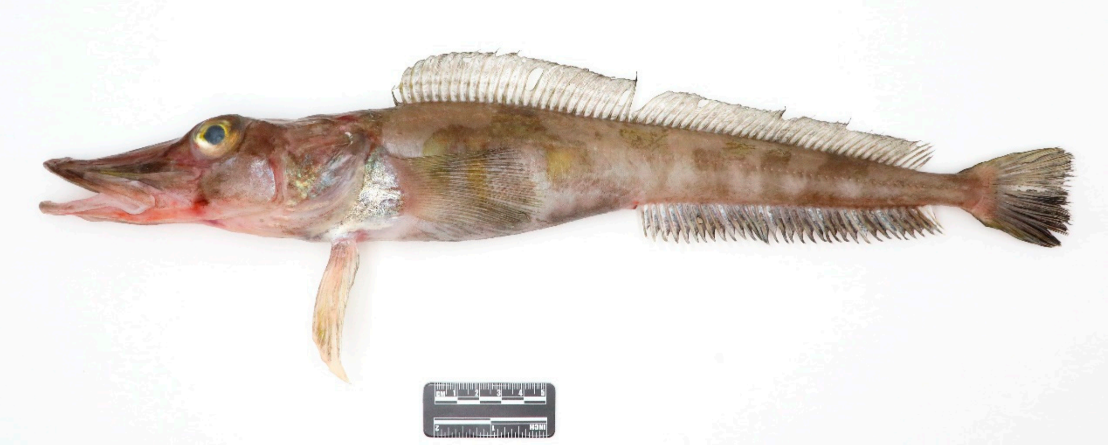

Figure S9 Morphological photos of *Cygnodraco mawsoni* Waite 1916.

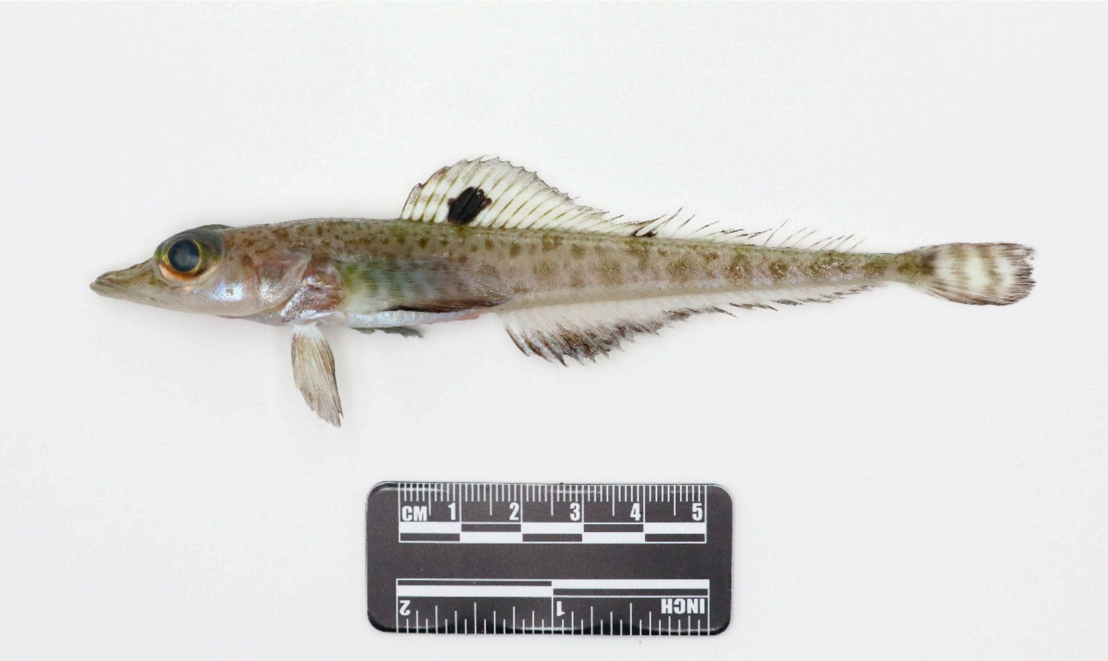

Figure S10 Morphological photos of *Prionodraco evansii* Regan 1914.

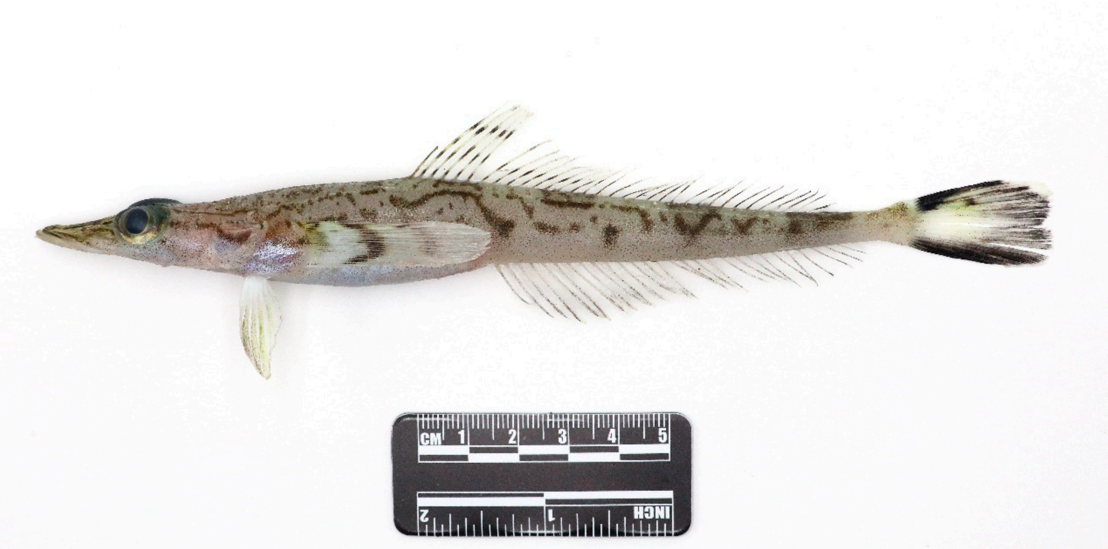

Figure S11 Morphological photos of *Racovitzia glacialis* Dollo 1900.

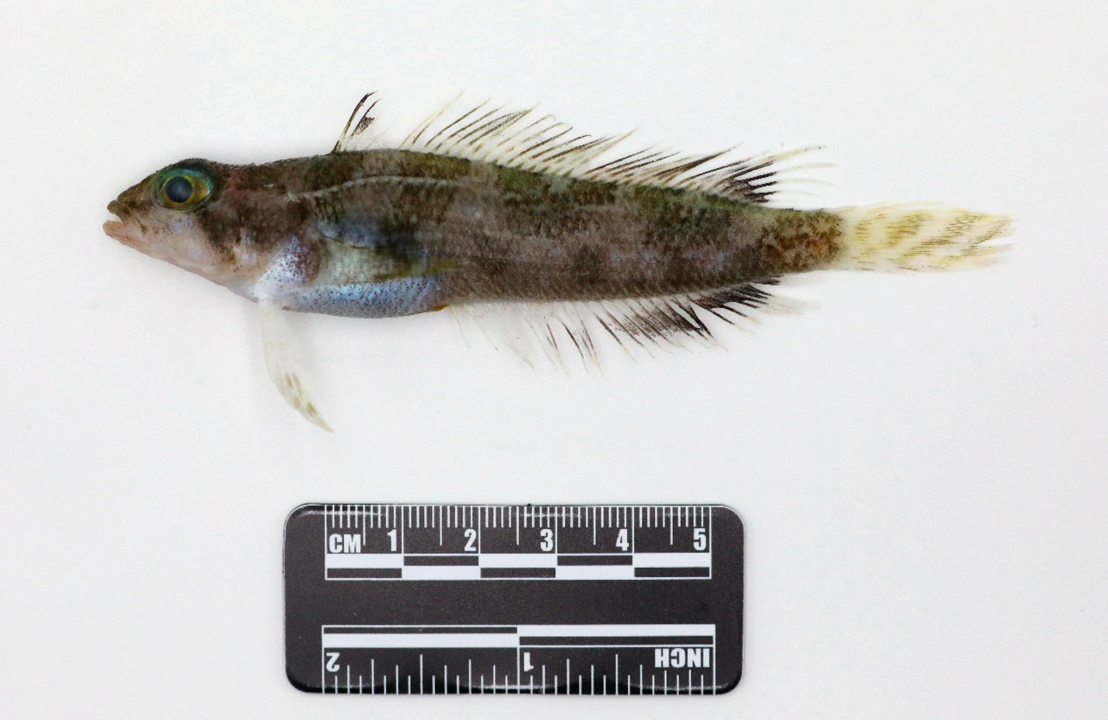

Figure S12 Morphological photos of *Trematomus scotti* (Boulenger 1907).
